# Are Psychosocial Factors Determinant in the Pain and Social Participation of Patients with Early Knee Osteoarthritis? A Cross-Sectional Study

**DOI:** 10.3390/ijerph18094575

**Published:** 2021-04-26

**Authors:** Ana Alabajos-Cea, Luz Herrero-Manley, Luis Suso-Martí, Juan Alonso-Pérez-Barquero, Enrique Viosca-Herrero

**Affiliations:** 1 Servicio de Medicina Física y Rehabilitación, Hospital La Fe, 46026 Valencia, Spain; alabajos.ana@gmail.com (A.A.-C.); luzherreromanley@gmail.com (L.H.-M.); juanalonsopb@gmail.com (J.A.-P.-B.); eviosca@gmail.com (E.V.-H.); 2 Grupo de Investigación en Medicina Física y Rehabilitación, Instituto de Investigación Sanitaria La Fe (IISLAFE), 46026 Valencia, Spain; 3 Departamento de Enfermería y Fisioterapia, Universidad CEU Cardenal Herrera, CEU Universities, 46115 Valencia, Spain

**Keywords:** osteoarthritis, early osteoarthritis, psychological factors, anxiety, depression, social participation

## Abstract

The main objective of this research is to determine the psychosocial differences between patients with knee pain or early osteoarthritis (EOA) and healthy subjects at risk of developing osteoarthritis. The secondary objective is to determine how psychosocial factors might influence pain and social participation in patients with EOA. A cross-sectional study was performed. Participants were divided according to the presence of pain or EOA. Pain intensity both at rest and walking, psychological variables such as anxiety and depression, and social participation were evaluated. A total of 105 participants were included (64 with knee pain and 41 without pain), with a mean age of 51.42 ± 5.92 (35 men and 70 women). Patients with knee pain had higher levels of anxiety (MD = −2.35; *p* < 0.01; *d* = 0.66) and depression (MD = −2.45; *p* < 0.01; *d* = 0.87), regardless of the presence of EOA. In addition, patients with higher depression levels had lower levels of social participation. The results revealed a relationship between the psychological variables, anxiety and depression, with knee pain and the onset of symptomatic OA, as well as an influence of depression levels on social participation. Improving these psychological characteristics may be useful in delaying the onset of symptomatic OA and enhancing social participation.

## 1. Introduction

Osteoarthritis (OA) is a joint disorder characterized by progressive degenerative changes [1,2] that may occur due to a wide variety of factors (e.g., post-traumatic, genetic, metabolic, biomechanical). Knee OA was typically associated with degenerative changes as a result of the progressive loss of articular cartilage, as well as subchondral bone changes, synovial inflammation, and meniscus degeneration [3,4]. However, knee OA presents with multifaceted symptoms and disruptions to daily life. Individuals with symptomatic knee OA report significantly worse function and overall health-related quality of life compared to age-matched healthy adults [5].

In this regard, multiple mechanisms can contribute to the perception of pain, such as nociception, neuropathic symptoms, psychological and personality factors, genetic influences, past painful experiences, comorbid conditions, and expectations related to future pain [6,7]. Among these factors, psychosocial factors such as depressive symptoms or higher levels of anxiety have been related to higher levels of pain and disability [8,9]. OA pain and function have been associated with greater depressive symptoms, and depressive symptoms are a robust predictor of knee pain worsening [10]. In people with knee OA, it has been described as the association with depressive symptoms, low self-efficacy in managing their OA symptoms, increased pain, catastrophizing, and increased fear of movement [11,12]. Additionally, the health benefits of a physically active lifestyle are well established in older adults with OA, where engaging in social activities might promote physical activity [13].

This relationship with psychological factors and social participation has been poorly studied for ‘early OA’ (EOA), before obvious structural or degenerative changes have been established in the joint [1,2,5]. A better understanding of this entity would lead to earlier diagnoses that could help stop the progression of the disease and develop better treatments [14].

Therefore, we believe that the identification of psychosocial factors related to the onset of symptomatology in patients with EOA could be an important way to detect and prevent the development of pain and symptomatology in patients with knee OA. The main objective of this research is to determine the psychosocial differences between patients with knee pain, EOA, and healthy subjects at risk of developing OA. The secondary objective is to identify the relationship between pain, psychosocial factors, and social participation in patients with EOA.

## 2. Materials and Methods

### 2.1. Study Design

A cross-sectional study with a non-probabilistic sample was performed. The design followed the international recommendations for strengthening the reporting of observational studies in epidemiology [15]. All the participants received an explanation of the study procedures, which were planned according to the ethical standards of the Declaration of Helsinki and approved by an Ethics Committee (CEIm La Fe 2017/0147). Written informed consent was obtained from all participants before their inclusion.

### 2.2. Participants

Subjects were recruited and followed at Hospital La Fe, Valencia, Spain, within the H2020 project OACTIVE. The design of the data collection protocol started in November 2017, and the recruitment and follow-up of participants started in July 2018 and lasted until February 2021.

The inclusion criteria for the pain group (PG) were (a) patient age greater than or equal to 40 years who experienced pain; (b) Kellgren and Lawrence (KL) 0–1. For the no pain group (NPG), the inclusion criteria were (a) patient age greater than or equal to 40 years, without pain or any knee symptoms; (b) Kellgren and Lawrence (KL) 0–1. The exclusion criteria were the same for both groups: (a) any cognitive disability that hindered the viewing of the audio-visual material; (b) illiteracy; (c) vomprehension or communication difficulties, (d) insufficient Spanish language comprehension to follow measurement instructions; (e) the presence of any rheumatic, autoimmune, or infectious pathology.

After the clinical evaluation, we reviewed the initial classification of every subject and refined it according to Luyten’s proposal for EOA classification:

(a) Patient-based questionnaires: knee injury and osteoarthritis outcome score: 2 out of the 4 KOOS subscales (pain, symptoms, function, or knee-related quality of life) need to score “positive” (≤85%).

(b) Patients should present joint line tenderness or crepitus during the clinical examination.

(c) X-rays: Kellgren and Lawrence (KL) grade 0–1 standing, weight bearing (at least 2 projections: PA fixed flexion and skyline for patellofemoral OA) [16].

### 2.3. Outcome Measures

#### 2.3.1. Descriptive, Demographic Data and Control Variables

We include in this section general demographic information such as gender, age, educational level, birth country, ethnicity, residency, marital status, and economic status [17,18]. Unhealthy behaviors such as smoking and drinking alcohol were registered. We also registered hormonal status in women and sports activities, defined as regular leisure activities. Finally, we collected weight and height data and calculated the participants’ BMI.

#### 2.3.2. Pain and Disability Variables

- Pain Intensity

The Visual Analogue Scale (VAS) was used to measure pain intensity before and after each treatment. The VAS is a 100 mm line with two endpoints representing the extreme states “no pain” and “the maximal pain imaginable”. It has been shown to have a good retest reliability (r = 0.94, *p* <.001) and a minimal detectable change of 15.0 mm [19,20]. Pain intensity was measured both at rest and while walking.

- Western Ontario and McMaster Universities Osteoarthritis Index (WOMAC)

This instrument is the most extensively used for the functional and symptomatic assessment of patients with osteoarthritis. The WOMAC questionnaire is self-administered and is used to assess patients who progress with hip and/or knee osteoarthritis. The questionnaire is a multidimensional scale composed of 24 items divided into 3 aspects: functional pain (consisting of 5 items), stiffness (2 items), and activities of daily life difficulties (17 items). Higher values mean poorer WOMAC subscales scores of pain and physical function. The Spanish version of the WOMAC questionnaire has adequate psychometric properties, presenting an index of internal consistency (a) of 0.82 for pain and 0.93 for physical function subscales [21].

#### 2.3.3. Psychological Variables

- Anxiety and Depression Symptoms

The Spanish version of the Hospital Anxiety and Depression Scale was used to assess the presence of depression and anxiety symptoms in the participants [22]. This scale includes 14 items, which are rated on a 4-point Likert-type scale. Two subscales assessed depression and anxiety independently. The internal consistency is 0.90 for the full scale, 0.84 for the depression subscale, and 0.85 for the anxiety subscale [22].

- Social Participation

The Maastricht Social Participation Profile included nine questions to determine the frequency and diversity of social involvement for older people with chronic diseases (Mars et al., 2009). The following nine items comprised social participation: ‘How often in the past four weeks have you (taken part in/been to): (1) a club, interest group or activity group, church, or other similar activity; (2) a cultural or educational event such as the cinema, theatre, museum, talk, or course; (3) eaten out; (4) gone out to a pub, café, or tearoom; (5) a public event; (6) an organized games afternoon or evening; (7) a day trip organized by a club or society; (8) committee work for a club, society, or another group; (9) any organized voluntary work? The response categories ranged from zero (‘not at all’) to three (‘more than twice a week’). We calculated the total scores for descriptive purposes, ranging from 0 to 27 (Cronbach’s αbaseline = 0.64; αfollow-up = 0.64), where higher scores equated to higher social participation [23].

### 2.4. Procedures

An information sheet with an explanation of the procedure and an informed consent form were given to all the participants. Once the subject had read the information from the study, they were allowed to ask any questions about its nature. The subjects that agreed to participate proceeded to fill in the sociodemographic questionnaire. Self-reported measures of disability, pain, and disability self-reported variables were then assessed. Finally, a physical examination was performed, including physical tests and motor and functional tests. The study protocol lasted for approximately one hour. In order to avoid fatigue affecting the physical tests, an interval of 3 min between tests was maintained. This procedure was identical for both groups.

### 2.5. Statistical Analysis

The sociodemographic and clinical variables of the participants were analyzed. The data were summarized using frequency counts, descriptive statistics, summary tables, and figures. The data analysis was performed using the Statistics Package for Social Sciences (SPSS 24, IBM Inc., Armonk, NY, USA). The categorical variables are shown as frequencies and percentages. The quantitative results are represented by descriptive statistics (confidence interval, mean difference (MD), and standard deviation). For all variables, the z-score was assumed to follow a normal distribution based on the central limit theorem because all the groups had more than 30 participants [24,25]. Student’s *t*-test was used for the group comparisons. Cohen’s *d* effect sizes were calculated for multiple comparisons of the outcome variables. According to Cohen’s method, the magnitude of the effect was classified as small (0.20–0.49), medium (0.50–0.79), or large (0.80).

The relationships between pain and disability measures, as well as between physical measurements, were examined using Pearson’s correlation coefficients. A Pearson’s correlation coefficient greater than 0.60 indicated a strong correlation, a coefficient between 0.30 and 0.60 indicated a moderate correlation, and a coefficient below 0.30 indicated a low or very low correlation [26].

## 3. Results

**Figure 1:**
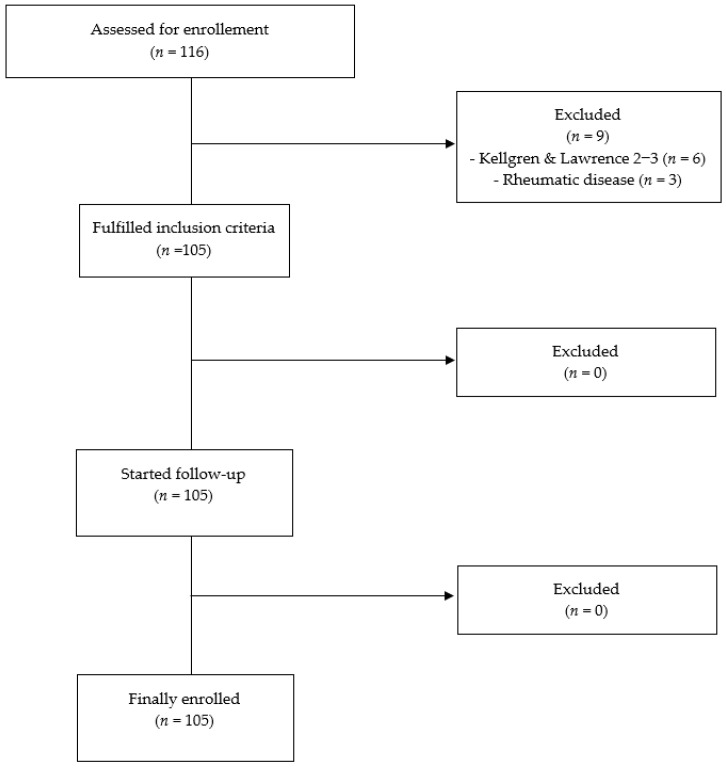
Study flow chart.

A total of 105 participants were included in the study, with a mean age of 51.42 ± 5.92 (35 men and 70 women). All the participants were finally enrolled and analyzed (Figure 1).

### 3.1. Pain Analysis

Participants were divided based on pain presence into two groups: PG (*n* = 64) and NPG (*n* = 41). The mean age for the PG was 51.77 ± 5.67 and 50.29 ± 6.38 for the NPG. There were no statistically significant differences between the groups in terms of age, gender, marital status, educational level, toxic habits, or hormonal status (Table 1).

Regarding the between-group comparisons, the *t*-test did not show statistically significant differences for WOMAC (0.18 ± 0.15 for PG and 0.21 ± 0.19 for NPG) and social participation (5.89 ± 3.44 for PG and 5.68 ± 2.73 for NPG). However, statistically significant differences were found for anxiety (MD = −2.35; *p* < 0.01; *d* = 0.66) and depression symptoms (MD = −2.45; *p* < 0.01; *d* = 0.87), with both showing higher values in the PG (Table 2).

### 3.2. OA Analysis

Participants were divided into two groups based on the EOA criteria: EOA (*n* = 54) and healthy subjects (HS) (*n* = 41). The mean age for EOA was 51.85 ± 5.72 and 50.49 ± 6.21 for HS. There were no statistically significant differences between the groups in terms of age, gender, marital status, educational level, toxic habits, or hormonal status.

Regarding the between-group comparisons, the *t*-test did not show statistically significant differences for WOMAC (0.21 ± 0.16 for EOA and 0.18 ± 0.17 for HS) and social participation (5.89 ± 3.23 for EOA and 5.73 ± 3.14 for HS). However, statistically significant differences were found for anxiety (MD = −2.29; *p* < 0.01; *d* = 0.65) and depression symptoms, with both showing higher values in patients with EOA (MD = −2.15; *p* < 0.01; *d* = 0.74). Additionally, VAS showed higher values for the EOA group, and statistically significant differences were also found for VAS rest (MD = −1.38; *p* = 0.03; *d* = 0.66) and VAS walking (MD = −2.42; *p* < 0.01; *d* = 0.99) (Table 3).

### 3.3. Psychosocial Variables Analysis

Differences between participants were analyzed according to their level of HAD depression. For this purpose, the median (median = 2) was calculated, and the participants were divided into a group with higher levels of depression (*n* = 64) and another with lower levels of depression (*n* = 51). There were no statistically significant differences between the groups in terms of age, gender, marital status, educational level, toxic habits, or hormonal status.

A statistically significant higher number (Chi-squared = 8.525; *p* = 0.004) of patients with EOA was found in the higher depression group (*n* = 37) compared to the HS group (*n* = 20). Similarly, the number of participants with lower levels of depression was lower in the EOA group (*n* = 17) compared to the HS group (*n* = 30).

**Figure 2:**
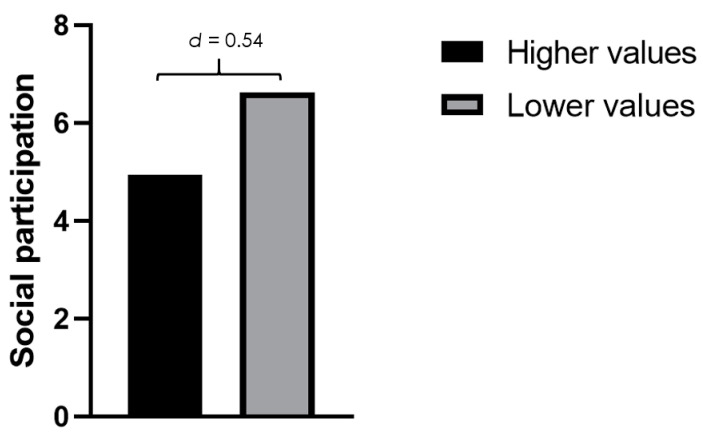
Social participation in participants with higher and lower levels of depression symptoms.

Regarding between-group comparisons, the *t*-test did not show statistically significant differences for WOMAC (0.19 ± 0.17 for the lower depression level group and 0.19 ± 0.15 for the higher depression level group). However, statistically significant differences were found for VAS rest, showing higher values in patients with higher depression values (MD = −1.39; *p* < 0.01; *d* = 0.65), VAS walking (MD = −1.87; *p* < 0.01; *d* = 0.68), and social participation, showing that patients with higher depression levels had lower levels of social participation (MD = 1.68; *p* = 0.04; *d* = 0.54) (Figure 2).

### 3.4. Correlation Analysis

Correlation analysis showed that higher levels of depression were correlated with higher levels of VAS at rest and walking, higher WOMAC, and less social participation, with a moderate correlation strength (r = 0.21 to 0.41; *p* < 0.05). In addition, higher anxiety levels were correlated with higher VAS at rest (r = 0.18; *p* < 0.05) and walking (r = 0.26; *p* < 0.01), but were not correlated with WOMAC levels and social participation (Table 4).

## 4. Discussion

The main objective of this research was to determine the psychosocial differences between patients with symptomatic EOA, asymptomatic EOA, and HS at risk of developing OA. Firstly, the results showed that patients with knee pain had higher levels of anxiety and depression, regardless of the presence of EOA. No differences were found in the WOMAC values, suggesting that psychological variables could have a stronger relationship with the occurrence of pain.

Knee pain is the primary complaint of those with knee OA and is associated with worse physical function, quality of life, and periarticular knee muscle function [27,28]. In this regard, numerous studies have shown discordance between the findings of imaging tests and the presence of painful symptomatology in patients with knee OA [29,30,31]. The main factor that could explain the discrepancy between degenerative signs and pain is that pain is a complicated condition with a multitude of components and pitfalls in measurement. The experience of pain is generated or modified by sensorial, emotional, and cognitive factors and, in turn, may induce higher levels of anxiety or depression in patients experiencing it [30].

Among these factors, the presence of anxiety or depression has been linked to a greater perception of pain and a lower quality of life [32]. This is a key aspect because pain is the main symptom of patients with OA and one of the main reasons for health care utilization [33]. In addition, it has been found that patients with OA who also present anxiety or depression have a worse perception of pain and lower functional capacity [34]. Our results show that anxiety and depression levels could be linked to the presence of knee pain and EOA, which could suggest a relationship between psychological factors and the onset of symptomatic OA. Furthermore, the correlation between depression and WOMAC suggests that using strategies to control these factors could improve the functional capacity of these patients, as suggested by previous studies [35].

Our secondary objective was to determine how psychosocial factors might influence pain and social participation in patients with EOA. In this regard, our results showed that social participation was not significantly influenced by pain or EOA. However, statistically significant differences were found among participants with higher levels of depression. Social participation is a frequently overlooked variable, yet it has previously been associated with higher mortality rates [36]. Previously, less social participation has been associated in patients with OA, and it has been suggested that it could be caused by poorer physical ability [37]. In this regard, it has been suggested that depression can be understood as a continuum of affective disorders developed by the influence of biopsychosocial factors. [38] According to our results, depression has also been associated with lower social participation in older adults, though it has not been previously studied in the OA population [39].

Finally, our data show that OA or pain could be less relevant factors than depression levels in relation to social participation, which underlines the need to include psychological interventions focused on reducing depressive symptoms in these patients from the beginning of the disease. Furthermore, they underline the need to rethink the clinical relevance of EOA. These results suggest that it may be more meaningful to assess pain or psychosocial factors in order to achieve better diagnostic and prognostic results. Further research on this matter is needed.

### Limitations

This study has some limitations that must be considered. The cross-sectional nature of this study makes it impossible to establish causality. Longitudinal studies are needed to reveal whether psychosocial factors are a cause or a consequence of knee pain in OA. In addition, it would have been interesting to assess more psychological variables as cognitive, emotional, and emotive factors may be related to the onset of symptoms in EOA and play a role in social participation outcomes [40]. Moreover, no corrections have been made for multiple comparisons, and this should be considered in the interpretation of the results. Finally, it should be noted that this is a single-center study with a limited sample size; thus, there could be selection bias. This could prevent us from extrapolating these findings to other clinical contexts. More studies are needed to further investigate the relationship between EOA and psychosocial factors.

## 5. Conclusions

The results of our study revealed a relationship between psychological variables, anxiety and depression, with knee pain and the onset of symptomatic OA. In addition, our data show that depression levels could be a relevant factor in relation to social participation in patients with EOA.

## Figures and Tables

**Table 1:** Descriptive and demographic data and control variables.

Variable	Pain Group (*n* = 64)	No-Pain Group (*n* = 41)	*p* Value
**Age** (years)	51.77 ± 5.67	50.29 ± 6.38	0.44
**BMI** (kg/m^2^)	26.94 ± 4.08	27.38 ± 3.75	0.59
**Gender**			0.12
Women	39 (60.9)	31 (75.6)	
Men	25 (39.1)	10 (24.4)	
**Marital status**			0.36
Single	13 (20.3)	5 (12.2)	
Married	39 (60.9)	29 (70.7)	
Divorced	9 (14.1)	5 (12.2)	
Widow	3 (4.7)	2 (4.9)	
**Education level**			0.55
Primary	10 (15.6)	7 (17.1)	
Secondary	25 (39.1)	13 (31.7)	
College	29 (44.3)	21 (51.2)	
**Birth Country**			0.67
Spain	59 (92.2)	64 (100)	
Other EU countries	2 (3.1)	0 (0)	
Other non-EU countries	3 (4.7)	0 (0)	
**Ethnicity**			0.89
White	62 (96.9)	40 (97.6)	
Black	0 (0)	1 (2.4)	
Hispanic American	2 (3.1)	0 (0)	
**Economic status**			0.34
Easy	17 (26.6)	10 (24.4)	
Fairly easy	35 (54.7)	25 (60.9)	
With some difficulties	9 (14.0)	6 (14.7)	
With great difficulties	3 (4.7)	0 (0)	
**Residency**			0.77
With family	52 (85.3)	32 (78.1)	
Independently	12 (18.7)	9 (21.9)	
**Hormonal status**			0.56
Postmenopausal	19 (48.7)	15	
Premenopausal	20 (51.3)	12	
**Alcohol**			0.89
Never	7 (10.9)	5 (12.2)	
Seldom	23 (35.9)	11 (26.8)	
1–2 times/month	13 (20.3)	9 (21.9)	
1–2 times/week	18 (28.2)	14 (34.2)	
1 time day	2 (3.1)	2 (4.9)	
More than 1 a day	1 (1.6)	0 (0)	
**Smoking**			0.52
Yes	11 (17.2)	4 (9.8)	
No	27 (42.2)	18 (43.9)	
Ex	26 (40.6)	19 (46.3)	

**Table 2:** Between-group comparisons.

Measures	Pain Group (*n* = 64)	No-Pain Group (*n* = 41)	Mean Difference (95% CI)	Effect Size (*d*)
**WOMAC**	0.18 ± 0.15	0.21 ± 0.19	0.03 (−0.05 to 0.11)	-
**HAD Anxiety**	5.62 ± 3.54	3.27 ± 3.55	−2.35 ** (−3.76 to −0.94)	0.66
**HAD Depression**	3.98 ± 3.12	1.54 ± 2.43	−2.45 ** (−3.59 to −1.31)	0.87
**Social Participation**	5.89 ± 3.44	5.68 ± 2.73	−0.21 (−1.47 to 1.05)	-

Note: ** *p* < 0.01.

**Table 3:** Between-group comparisons.

Measures	EOA (*n* = 54)	HS (*n* = 51)	Mean Difference (95% CI)	Effect Size (*d*)
**WOMAC**	0.21 ± 0.16	0.18 ± 0.17	−0.03 (−0.09 to 0.05)	-
**VAS Rest**	2.17 ± 2.53	0.78 ± 1.87	−1.38 * (−2.25 to −0.52)	0.62
**VAS Walking**	3.17 ± 2.86	0.75 ± 1.91	−2.42 ** (−3.37 to −1.48)	0.99
**HAD Anxiety**	5.80 ± 3.69	3.50 ± 3.39	−2.29** (−3.67 to −0.92)	0.65
**HAD Depression**	4.06 ± 3.19	1.90 ± 2.59	−2.15 ** (−3.29 to −1.02)	0.74
**Social Participation**	5.89 ± 3.23	5.73 ± 3.14	−0.16 (−1.39 to 1.07)	-

EOA: early osteoarthritis; healthy subjects. Note: ** *p* < 0.01; * *p* < 0.05.

**Table 4:** Correlation analysis.

Variable	*HAD Anxiety*	*HAD Depression*
***VAS Rest***	0.18 *	0.32 *
***VAS Walking***	0.26 **	0.41 **
***WOMAC***	0.06	0.23 *
***Social Participation***	−0.14	−0.21 *

Note: ** *p* < 0.01; * *p* < 0.05.

## Data Availability

Not applicable.
